# Efficacy of *Melaleuca alternifolia* and *Pelargonium graveolens* Oils Against *Staphylococcus aureus* and *Staphylococcus epidermidis*: An In Vitro Study

**DOI:** 10.3390/microorganisms13112467

**Published:** 2025-10-29

**Authors:** Ntombokhanyo Mbanjwa, Gaofetoge Lenetha, Refilwe Molatlhegi, Ntelekwane George Khasapane

**Affiliations:** 1 Department of Life Science, Central University of Technology, Free State, Bloemfontein 9300, South Africa; okuhleoluhle35@gmail.com; 2 Division of Student Learning and Development, University of the Free State, Bloemfontein 9300, South Africa; setlharegg@ufs.ac.za; 3 Department of Medical Microbiology, School of LMMS Medical School, College of Health Sciences, University of KwaZulu-Natal, Durban 4041, South Africa; molatlhegir1@ukzn.ac.za

**Keywords:** antimicrobial activity, antibiotic-resistant bacteria, essential oils

## Abstract

The rise of antibiotic-resistant bacteria around knee implants significantly diminishes patients’ quality of life and mobility, necessitating innovative solutions to combat infections. This study explores the antimicrobial efficacy of tea tree (*Melaleuca alternifolia*) and geranium (*Pelargonium graveolens*) essential oils against *Staphylococcus aureus* and *Staphylococcus epidermidis*. Utilizing bioassay preparation methods and minimal inhibitory concentration (MIC) assays, we found that tea tree oil notably inhibited *S. aureus* growth, while Geranium oil effectively targeted *S. epidermidis*. Scanning and transmission electron microscopy revealed substantial morphological alterations in both bacterial strains following treatment with the essential oils. Twenty compounds were identified by GC/MS chemical profiling in tea tree oil, with α-pinene (21.6%), γ-terpinene (21.1%), and terpinen-4-ol (17.3%) being the main ingredients. Forty compounds were found in Geranium oil, with citronellol (42.2%), geraniol (30.5%), and linalool (9.8%) constituting the majority. Our findings suggest that incorporating these essential oils into orthopaedic implants could significantly enhance their antibacterial effectiveness, offering a promising alternative to traditional antibiotic treatments and potentially reducing infection rates associated with knee implants. This research not only contributes to the understanding of natural antimicrobial agents but also paves the way for their practical application in clinical settings, addressing the critical challenge of antibiotic resistance.

## 1. Introduction

Knee implants are commonly used to replace damaged joints; however, the presence of foreign materials increases the risk of bacterial adhesion, particularly from staphylococci, which are responsible for a significant proportion of prosthetic joint infections (PJIs). Notably, *Staphylococcus aureus* accounts for 22–23.6% and *Staphylococcus epidermidis* for 19–37.5% of these infections [1,2]. The formation of biofilms on implant surfaces complicates treatment, as bacteria within these structures exhibit reduced metabolic activity, increased resistance to antimicrobial agents, and enhanced virulence due to the protective extracellular matrix they produce [3,4,5,6,7,8]. As a result, biofilm-associated infections pose substantial therapeutic challenges, leading to increased hospital admissions and treatment failures [9]. The emergence of drug-resistant bacteria further exacerbates this public health concern [10], highlighting the urgent need for innovative solutions to combat implant-related infections.

To address this challenge, researchers have turned to the antimicrobial properties of essential oils (EOs) derived from various medicinal plants. EOs, which are concentrated aromatic extracts, have been shown to possess diverse bioactive compounds with antibacterial, anti-inflammatory, and antioxidant properties [11,12,13,14,15,16,17,18,19]. Recent studies indicate that EOs, such as tea tree and geranium, can effectively inhibit the growth of antibiotic-resistant bacteria commonly associated with knee implants, thereby offering a promising alternative to conventional antibiotics [20,21]. The principal components of geranium oil, including geraniol and citronellol, contribute to its antimicrobial efficacy [22], while tea tree oil, rich in terpinen-4-ol, is recognised for its potent antibacterial and antiseptic properties [23].

Despite extensive research on the antibacterial activities of these EOs in various global contexts [24,25,26,27], there remains a notable gap in their evaluation within the African continent. Thus, this study aims to fill this gap by characterising tea tree and geranium oils through gas chromatography–mass spectrometry (GC-MS), assessing their antimicrobial activity against *S. aureus* and *S. epidermidis* using a bioassay preparation method and minimum inhibitory concentrations, and examining their effects on bacterial morphology using scanning and transmission electron microscopy. This research not only contributes to understanding the potential applications of EOs in preventing implant-related infections but also paves the way for their integration into orthopaedic materials, potentially enhancing the clinical efficacy of knee implants.

## 2. Materials and Methods

### 2.1. Essential Oils

*Melaleuca alternifolia* and *Pelargonium graveolens* oils were purchased from a local supplier in Bloemfontein (29.1129° S, 26.2149° E), which is situated in the Free State province, South Africa.

### 2.2. Microorganisms

Strains of *Staphylococcus aureus* ATCC 25,923 and *Staphylococcus epidermidis* ATCC 12,228 were acquired from Thermo Fisher Scientific, located in Johannesburg, South Africa. The strains were sub-cultured weekly on plate count agar (PCA) (Merck, Johannesburg, South Africa) at 37 °C for the duration of 24 h. Thereafter, the cultures were then adjusted to a turbidity of 0.5 McFarland, which is approximately 10^8^ colony-forming units (CFU) per mL for further analysis [28] and 10^5^ CFU/mL for the microdilution method, using sterile saline solution.

### 2.3. Chromatographic Analyses

Essential oils derived from *Melaleuca alternifolia* and *Pelargonium graveolens* were purchased from local sources. These essential oils were classified based on the technique described by Kirbaslar et al. [29]. Hexane was used to briefly dissolve the oils (10% hexane) then administered a Finnigan Focus gas chromatograph (GC) (Thermo Fisher Scientific, Johannesburg, South Africa) at a split ratio of 50:1. The temperature of the injector was set at 230 °C. An AB-1MS (30 m × 0.25 μm) capillary column was installed in the GC. The carrier gas utilised was helium, which flowed at a steady 1 mL min^−1^. The programme’s temperature was set at 240 °C for 4 min before being elevated to 5 °C min^−1^ to 200 °C after which it was held at 200 °C for 1 min and then elevated to 220 °C, where it was kept for 10 min. The oils were subjected to mass analysis utilising a Finnigan Focus DSQ mass spectrometer ((Thermo Fisher Scientific, Johannesburg, South Africa). The ion source was at 250 °C with an ionisation voltage of 70 eV and mass scan range of 50–650 amu. Individual GC peaks and mass spectra were found by searching commercial libraries. After the identification procedure, MS data was expertly matched.

### 2.4. Bioassay Preparation

A method for quantitative microbiological bioassay was utilised to evaluate the antibacterial efficacy of essential oils on bacterial growth. To make inocula, cultures were cultivated overnight at 37 °C. Bacterial density was then adjusted to around 10^8^ colony-forming units (cfu/mL) per ml for bioassay preparation. Bacterial strains used in bioassay were regrown on Petri dishes and incubated for 24 h at 37 °C. Next, each bacterium was immersed in sterilised distilled water and 0.1 mL was smeared on PCA (0.5% m·v^−1^ agar). This created a lawn that was uniform over the entire agar surface [30]. Then, a well (diameter and depth of 0.5 cm) was built in the centre of the Petri dish and 46 μL of essential oils together with ethanol were added The plates were all kept in the incubator at 37 °C until regions of growth of different textures were noticeable (after 24 h). To prevent essential oils from evaporating from the plates, the oils tested were diffused in the agar prior to incubation. After that the inhibition growth zones were detected and measured in diameters (mm).

### 2.5. Microdilution Assay

Microdilution essay was conducted, the bacterial strains’ inoculum were prepared in Mueller-Hinton broth (MHB) for about 24 h, and were modified to meet 0.5 McFarland turbidity standard (roughly 1.5 × 10^8^ cfu/mL). Every suspension of bacteria was then dispersed into a 96-well sterile microtiter plate as displayed Abidin et al. [31] and Desam et al. [32]. The initial row of holes was filled with each essential oil and dilutions in series were carried out to achieve the desired concentrations of 12.5, 6.25, 3.125, 1.56, 0.8, 0.4, 0.2 and 0.1 μg/mL. The plate was closed by using sealer of sterile plate and then grown under controlled conditions in the laboratory at 37 °C overnight. To demonstrate development of growth in a 24 h period, the test was performed two times for each concentration in duplicate. Following this, each well was then filled with p-iodonitrotetrazolium violet (INT) of about 20 µL. The plate was then put in the incubator at 37 °C for 20 min. Growth was seen by a shift in colour from pink to violet.

### 2.6. The Manufacturing Method of the Experimental Ti6Al4V Knee Samples

Titanium knee implants were created utilising the EOS M280 direct metal laser sintering (DMLS) technology within the laser powder bed fusion (LPBF) framework [33,34]. The process utilised spherical argon-atomized Ti6Al4V (ELI) powder from TLS Technik, maintaining an argon atmosphere with oxygen levels between 0.07% and 0.1%. Important production specifications were a zigzag scanning strategy, a laser power of 170 W, a scanning speed of 1.25 m/s, a hatch distance of 80 µm, and a powder layer thickness of 30 µm. [33,34]. Following manufacturing, the samples were separated by electrical discharge machining, stressed for three hours at 650 °C in an argon atmosphere, and ultrasonically cleaned to get rid of any remaining powder.

### 2.7. Scanning Electron Microscopy (SEM) on the Titanium Implant Materials

Scanning electron microscopy (SEM) (Dearborn Road Peabody, MA) was employed to evaluate the resulting structural alterations due to the antibacterial action of geranium and tea tree essential oils on *S. aureus* and *S. epidermids* on the titanium implant’s surface. Getting cells ready for assessment using SEM was completed in accordance with the guidelines suggested by Ncango et al. [35]. Bacterial cells, both treated and untreated on the titanium implant material’s surface (utilising the microdilution assay method) were primarily fixed utilising 3% *v*/*v* of a sodium phosphate-buffered glutaraldehyde solution at pH 7.0 0 and a similarly buffered solution (1% *m*/*v*) of osmium tetroxide for about an hour. In order to dehydrate the titanium implant material, a succession of ethanol solutions was graded (30%, 50%, 70%, 90%, and 100% for 20 min for each solution, and the 100% dehydration was carried twice for 1 h). After that, the ethanol-dehydrated titanium implant material was heated at 40 °C and followed by sputter coating with uranium for 30 min to produce electrical conductivity. Next, the coated implant was inspected utilising SEM. To examine the structural alterations brought about by essential oils on the bacterial cells, pictures were obtained.

### 2.8. Transmission Electron Microscopy (TEM) on the Titanium Implant Materials

Transmission electron microscopy (Phillips, Amsterdam, The Netherlands) was utilised to evaluate the structural modifications that had happened because to the antibacterial action of geranium and tea tree essential oils on *S. aureus* and *S. epidermids* on the titanium implant material’s surface. Getting the cells ready for assessment utilising transmission electron microscopy was completed in accordance with to the suggested procedures van Wyk and Wingfield [36]. Bacterial cells, both treated and untreated on the titanium implant material’s surface (utilising the microdilution assay procedure) were fixed primarily utilising 3% *v*/*v* of a sodium phosphate-buffered glutaraldehyde solution at pH 7.0 0 and a similarly buffered solution (1% *m*/*v*) of osmium tetroxide for about an hour. In order to dehydrate the titanium implant material, a succession of ethanol solutions was graded (30%, 50%, 70%, 90%, and 100% for 20 min for every solution, and the 100% dehydration was carried twice for 1 h). After that, the ethanol-dehydrated titanium implant material was heated at 40 °C and followed by sputter coating with uranium for 30 min to produce electrical conductivity. Next, the coated implant was inspected utilising transmission electron microscopy. To examine the structural alterations brought about by essential oils on the bacterial cells, pictures were obtained.

### 2.9. Statistical Analysis

To compare the mean effects of the oils between species (e.g., *S. epidermidis* vs. *S. aureus*), we ran the two-sample *t*-test using Microsoft ExcelVersion 2024.

## 3. Results and Discussion

### 3.1. Chemical Composition of the Selected Essential Oils

The chemical constituents of tea tree and geranium essential oils are crucial to understanding their modes of action, particularly in the context of their antibacterial properties. The study presents the detailed chemical profiles of these essential oils, as determined through gas chromatography–mass spectrometry (GC-MS). The analysis of *Melaleuca alternifolia* (tea tree essential oil) revealed the presence of 20 distinct compounds, with notable components including α-pinene (21.64%), γ-terpinene (21.09%), terpinen-4-ol (27.31%), limonene (12.3%), and cymene (10.1%) (Table 1). In contrast, *Pelargonium graveolens* (geranium essential oil) exhibited a more complex profile, identifying 27 different compounds. Dominant constituents included citronellol (42.2%), geraniol (21.7%), isomenthone (15.3%), citronellyl formate (14.7%), linalool (19.8%), geranyl formate (16.3%), and trans-Calamenene (13.2%) (Table 2).

According to Badr et al. [37] terpinen-4-ol, which constitutes a significant portion of tea tree essential oil at 41.11%, possesses notable antibacterial properties that enable it to disrupt the morphological and functional integrity of bacterial membranes. The current study supports this assertion, identifying terpinen-4-ol as a key component of tea tree essential oil at 27.31%, which directly correlates with its ability to compromise the membranes of *S. aureus* and *S. epidermidis*. Other notable constituents of tea tree essential oil, including α-pinene, γ-terpinene, limonene, and cymene, have also been associated with antibacterial and anti-inflammatory properties [38].

Fayoumi et al. [39] (conducted a GC-MS analysis of geranium essential oil, identifying citronellol (30.5%) and geraniol (12.8%) as major components that contribute to cell wall disruption, loss of cellular contents, and overall bacterial deformation upon exposure to geranium essential oil. The present study corroborates these findings, identifying similar major components in geranium essential oil.

Moreover, Kamel et al. [38] highlighted that the presence of citronellol and geraniol in geranium essential oil endows it with antifungal, anti-inflammatory, and antimicrobial properties. The ability of geranium essential oil to reduce inflammation can alleviate joint pain and enhance mobility in affected areas. This study further confirms the presence of both citronellol and geraniol in geranium essential oil, which may explain its effectiveness in inhibiting the growth of *S. aureus* and *S. epidermidis*. Notably, over 40% of the components in geranium essential oil consist of alcohol-based compounds that exhibit antibacterial characteristics, as documented by Bigos et al. [27]. Geranium essential oil is effective against strains of *S. aureus* and *S. epidermidis* with various drug-resistant mechanisms, making it a viable candidate for inclusion in the treatment of knee infections.

The use of essential oils such as tea tree and geranium in addressing human infections caused by multidrug-resistant bacterial strains presents an innovative alternative to synthetic pharmaceuticals. However, it is crucial to recognise that the percentage compositions of the identified constituents in essential oils can vary significantly due to factors such as plant growth conditions, genetic variability, chemical forms, harvesting seasons, and nutritional status of the plants.

### 3.2. Antimicrobial Properties of Essential Oils

This study employed the bioassay method to evaluate the antimicrobial activity of tea tree and geranium essential oils against antibiotic-resistant strains of *S. aureus* and *S. epidermidis*. The bioassay was designed to identify essential oils exhibiting inhibition diameters of at least 20 mm. Following this, the essential oils were applied to the surfaces of titanium knee implants to assess their efficacy in inhibiting bacterial growth. Both tea tree and geranium essential oils demonstrated significant inhibitory effects on the growth of *S. aureus* and *S. epidermidis*. Further investigations were conducted to determine the MICs of these essential oils and to examine the structural changes they induced in bacterial cells. The findings of this study indicate that tea tree and geranium essential oils may be effectively utilised on the surfaces of titanium knee implants to prevent bacterial colonisation. However, further research is needed regarding essential oils’ adherence to titanium knee implants, to be precise.

#### 3.2.1. Initial Key Findings

The results of the inhibition zones are presented in Table 3. The essential oils exhibited varying degrees of efficacy, which may be attributed to the specific modes of action of each oil against the bacterial species examined. As noted by Badr et al. [37], terpinen-4-ol, a key component of tea tree essential oil, along with citronellol and geraniol found in geranium essential oil, may be responsible for the observed antimicrobial activity against *S. aureus* and *S. epidermidis* [37,40].

Tea tree essential oil is recognised for its broad spectrum of activity, which includes antifungal, antiviral, and antiprotozoal properties [37]. The components of tea tree and geranium essential oils demonstrated inhibitory effects, which likely contribute to their ability to disrupt the morphological and functional integrity of bacterial membranes [37]. This study confirmed that both tea tree and geranium essential oils effectively inhibit the growth of *S. aureus* and *S. epidermidis*, as shown in Table 1.

Though the *p*-value suggests a trend (with *S. aureus* exhibiting greater mean inhibition zones than *S. epidermidis*), the difference is not statistically significant at the standard significance level of α = 0.05.

**Figure 1:**
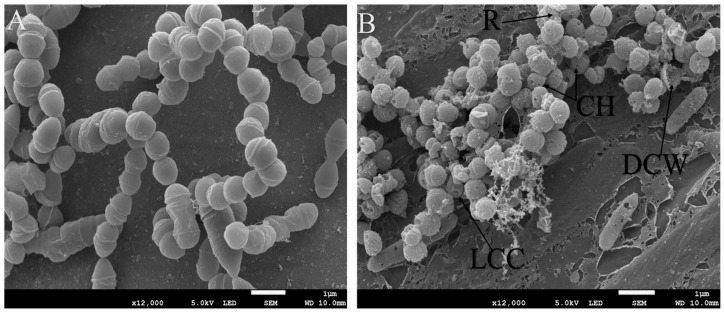
SEM picture (**A**) of titanium’s’ implant presenting *S. aureus* cell’s control. Picture (**B**) present *S. aureus* cells which are treated by *Geranium* essential oil. Demonstrating a decrease in content of bacteria cell (LCC), and a damaged to cell’s wall (DCW), cell holes (CH) and roughness (R).

Specifically, tea tree essential oil exhibited the most substantial inhibitory effect on *S. aureus*, with the largest inhibition zone measuring 39.1 mm (Table 1). Geranium essential oil also demonstrated significant antimicrobial activity against *S. aureus*, with inhibition zones of 32.8 mm. Furthermore, geranium essential oil effectively inhibited the growth of *S. epidermidis*, exhibiting an inhibition zone of 24.6 mm, while tea tree essential oil showed an inhibition zone of 20.5 mm against the same strain (Table 1 and Figure 1). Notably, a study by Abdelhamed et al. [41] corroborated these findings, confirming that tea tree essential oil can prevent the growth of *S. epidermidis*.

Overall, these results underscore the potential of tea tree and geranium essential oils as effective antimicrobial agents, highlighting their applicability in preventing infections associated with knee implants.

#### 3.2.2. Further Testing and Results

The findings from the bioassay preparation revealed that both essential oils exhibited significant antimicrobial activity, highlighting their potential for use as coatings on titanium knee implants to assess their efficacy in inhibiting bacterial growth on the implant surfaces. In this phase of the study, both tea tree and geranium essential oils were selected for application on the titanium knee implants to prevent the growth of *S. aureus* and *S. epidermidis*. However, before these oils were utilised on the implant surfaces, their MICs against the bacterial strains were determined through a microdilution assay.

Notably, each essential oil demonstrated varying responses against the different bacterial strains tested, a phenomenon that can be attributed to the distinct chemical compositions inherent to each oil. This variability emphasises the necessity of considering the specific interactions between essential oil constituents and bacterial species when developing antimicrobial coatings for biomedical applications.

To further evaluate the concentrations at which geranium and tea tree essential oils inhibit the growth of *S. aureus* and *S. epidermidis*, a microdilution assay was conducted. The results indicated that both essential oils exhibited strong antibacterial activity. Specifically, the MIC value for geranium essential oil against *S. aureus* was determined to be 0.4 μg/mL (Table 4), which aligns closely with findings reported by Mahboubi et al. [42], who noted that geranium essential oil demonstrated potent activity against *S. aureus* isolates and multidrug-resistant strains, with MIC values ranging from 0.25 to 2.50 μg/mL. Similarly, Hsouna and Hamdi [43] recorded MIC values of 0.312 and 0.625 μg/mL for geranium essential oil against *S. aureus*.

Elghali et al. [44] suggested that the antibacterial activity of Geranium essential oil may be attributed to its chemical composition and the concentration of active molecules present. The robust antibacterial action of Geranium is likely linked to the abundance of geraniol and citronellol. Tea tree oil, containing components such as α-pinene, γ-terpinene, terpinen-4-ol, limonene, and cymene, which were also identified in this study (Table 1), has been individually associated with antibacterial and anti-inflammatory properties [38].

Building on the findings presented, further analysis was warranted to investigate the antimicrobial potential of *Melaleuca alternifolia* (Tea tree) and *Pelargonium graveolens* (Geranium) essential oils against bacterial colonisation on titanium knee implants. These essential oils have previously been utilised to address bacterial infections that colonise the surfaces of titanium implant materials, which can lead to early implant failure. It is well-documented that coating implant materials with antibiotics, antiseptics, or other antimicrobial agents can effectively inhibit bacterial growth on their surfaces [45,46]. As such, the current study aimed to explore the application of essential oils as a strategy to prevent bacterial colonisation on titanium knee implants.

The antimicrobial activity of *Melaleuca alternifolia* and *Pelargonium graveolens* essential oils on titanium knee implant materials was qualitatively assessed by determining the Minimum Inhibitory Concentrations (MICs) of these oils against *S. aureus* and *S. epidermidis*. The microdilution method was employed for this purpose. Titanium implant materials were immersed in solutions containing the respective essential oils to evaluate their MICs. The results indicated that both Geranium and Tea tree essential oil solutions exhibited an MIC of 6.25 μg/mL against *S. aureus*, while a lower MIC of 3.13 μg/mL was observed for both oils against *S. epidermidis*.

These essential oils were subsequently applied at their determined MICs to investigate the morphological changes they induced both externally and internally in bacterial cells. Scanning Electron Microscopy (SEM) and Transmission Electron Microscopy (TEM) were utilised to conduct a thorough examination of the structural alterations imposed by the essential oils on the isolates of *S. aureus* and *S. epidermidis*.

Additionally, Tea tree essential oil exhibited antibacterial activity against *S. aureus* with an MIC value of 0.4 μg/mL. Furthermore, Geranium essential oil showed a MIC value of 0.1 μg/mL and Tea tree 0.2 μg/mL against *S. epidermidis*. These MIC results collectively indicate that both Geranium and Tea tree essential oils possess potent antibacterial activity against both *S. aureus* and *S. epidermidis*, as summarised in Table 4 and Table 5, highlighting their relevance in the context of knee implants.

Elghali et al. [44] suggested that the antibacterial activity of Geranium essential oil may be attributed to its chemical composition and the concentration of active molecules present. The robust antibacterial action of Geranium is likely linked to the abundance of geraniol and Tea tree oil, containing components such as α-pinene, γ-terpinene, terpinen-4-ol, limonene, and cymene, which were also identified in this study (Table 1 and Table 2), has been individually associated with antibacterial and anti-inflammatory properties [38].

The rising prevalence of knee implants has led to a significant public health concern, particularly due to the increasing rates of antibiotic resistance [47]. For instance, *Staphylococcus aureus* has developed resistance to both methicillin and penicillin, rendering these antibiotics ineffective by limiting the formation of cell wall openings necessary for their action. While antibiotic molecules typically enter Gram-positive bacteria through diffusion via external membrane porins, a reduction in the number of porin channels impedes antibiotic entry into the cell [11,48,49]. Furthermore, bacteria possess efflux pumps that actively expel antibiotics, including groups such as Tetracyclines, Lincosamides, Phenicols, and Lipopeptides [13].

These efflux pumps operate at a rate commensurate with the influx of antibiotics, effectively expelling them before they can reach their intracellular targets [11,49]. The exterior membranes of Gram-positive bacteria act as barriers, preventing antibiotics from penetrating bacterial cells during their evolutionary adaptations. Consequently, microorganisms have developed resistance mechanisms that enable them to survive and proliferate in the presence of antibiotics, thereby limiting the effectiveness of antimicrobial agents. In contrast, essential oils have demonstrated the ability to inhibit the growth of many bacterial and fungal pathogens. Essential oils can penetrate the bacterial cell wall, target the cytoplasm and membrane, and induce structural changes that inhibit pathogen growth [11].

To assess the effects of Geranium and Tea tree essential oils on *S. aureus* and *S. epidermidis*, Scanning Electron Microscopy (SEM) and Transmission Electron Microscopy (TEM) techniques were employed. The SEM results revealed observable morphological changes, including slight roughness (R) of the cell surfaces, decreased cellular content (LCC), and damage to the cell walls (DCW) (Figure 1A,B). These figures illustrate that the application of Geranium and Tea tree essential oils adversely affected the cell wall structures of both treated *S. aureus* and *S. epidermidis*. This indicates a significant impact of the essential oils on the structural integrity of these antibiotic-resistant bacteria, as evidenced by the control cells, which exhibited no such changes.

**Figure 2:**
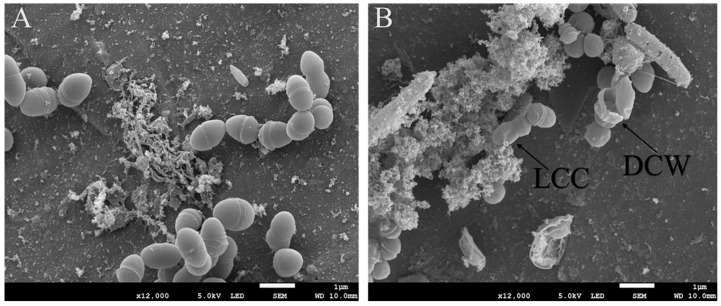
SEM picture (**A**) of titanium’s’ implant presenting *S. epidermidis* cell’s control. Picture (**B**) shows *S. epidermidis* cells treated by Tea tree essential oil. Demonstrating a decrease in cell’s content (LCC) and a damaged to cell’s wall (DCW).

Furthermore, Kamel et al. [38] have previously demonstrated the efficacy of Tea tree and Geranium oils in inhibiting the growth of *S. aureus*, which is commonly associated with knee implant infections. This study further corroborates the effectiveness of essential oils in combating bacterial infections in the knee. Additionally, prior investigations by Nguyen et al. (2023) indicated that Tea tree oil disrupts bacterial membranes and acts as a membrane permeabilizer, compromising the bacteria’s ability to regulate their chemiosmotic processes for both Gram-positive and Gram-negative organisms [38]. Nguyen et al. [50] also reported that Tea tree essential oil caused cell membrane disruption in *S. epidermidis*, leading to cell death. Correspondingly, this study also demonstrated the membrane disruption of both *S. aureus* and *S. epidermidis* by Tea tree essential oil, as illustrated in Figure 2.

**Figure 3:**
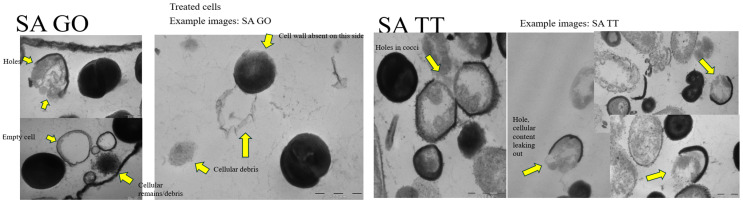
Treated *S. aureus* (SA) cell’s with tea tree (TT) and geranium (GO) essential oils.

**Figure 4:**
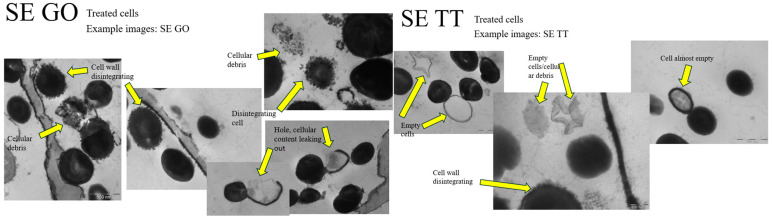
Treated *S. epidermidis* (SE) cell’s with tea tree (TT) and geranium (GO) essential oils.

The TEM results, presented in Figure 3 and Figure 4, revealed drastic alterations in the cellular structure of *S. aureus* and *S. epidermidis* following exposure to Tea tree and Geranium essential oils. Notable changes included damaged cell walls, the formation of holes, and the depletion of cellular contents, ultimately resulting in cell death. The observed intercellular leakage and morphological alterations in the treated cells confirm that Tea tree and Geranium essential oils significantly impact the structural integrity of the cell walls of *S. aureus* and *S. epidermidis*. Thus, it can be argued that these essential oils possess the potential to serve as effective antimicrobial agents against antibiotic-resistant strains of *S. aureus* and *S. epidermidis* associated with knee implants.

## 4. Conclusions

This study demonstrates that essential oils, specifically tea tree and geranium, exhibit potent antimicrobial activities against *S. aureus* and *S. epidermidis* isolates. These findings underscore the potential of incorporating these natural products into orthopaedic applications, particularly in the development of coatings for titanium implants to prevent infections associated with knee surgeries. The mechanism of action of these essential oils, which includes compromising cell walls and disrupting cytoplasmic membranes, enhances their effectiveness as antimicrobial agents. Future research should focus on investigating the application of these essential oils in preventative strategies and further exploring their adhesion properties to titanium surfaces. This work not only contributes to the growing body of evidence supporting the use of essential oils in clinical settings but also opens new avenues for enhancing the longevity and safety of orthopaedic implants.

## Figures and Tables

**Table 1:** Relative proportions of the tree tea oil constituents by GC/MS analysis.

Compound	Relative (%)	RI
α-pinene	21.64	937.1
γ-terpinene	21.09	1063.5
limonene	9.37	1033.9
γ-Eudesmol	8.4	1650
Geranyl valerate	8.0	1630
Citronellyl tiglate	5.6	1692
Geranyl propionate	4.5	1476
Trans-Ascaridol glycol	4.2	1088
limonene dioxide	2.8	987
α-Terpineol	2.2	1204.4
Globulol	1.9	1090
β-Myrcene	1.1	990.1
Ethanone,1-(6-methyl-7-oxabicyclo [4.1.0]hept-1-yl)-	1.0	978
3-Thujene	0.7	980.5
1,4-dihydroxy-p-menth-2-ene	0.5	1118
β-Pinene	0.4	979
Sobrerol	0.3	1087
Geranyl tiglate	0.1	1675
Cymene	0.1	1015
***p*** **-value**	**1.86123 × 10^−5^**	

RI: Retention Index.

**Table 2:** Relative proportions of the geranium oil constituents by GC/MS analysis.

Compound	Relative (%)	RI
Citronellol	22.4	1217
Geraniol	5.7	1243
Isomenthone	5.3	1144
citronellyl formate	4.0	1261
Linalool	2.18	1086
geranyl formate	2.0	1283
Phenylethyl tiglate	2.0	1554
geranyl tiglate	1.4	1675
Neryl formate	1.2	1264
Geranial	1.1	1246
Selina-4(15),7(11)-diene	1.1	1530
α-Pinene	1.0	929
Geranyl propionate	1.0	1452
α-Terpineo	0.9	1173
δ-Cadinene	0.9	1515
Geranyl butyrate	0.7	1537
Alloaromadendrene	0.6	1459
Isomenthone	0.4	1144
Nerol	0.3	1220
β-Pinene	0.2	979
Zonarene	0.2	1518
10-epi-γ-eudesmol	0.2	1613
Isomenthol	0.1	1168
***p*** **-value**	**2.02 × 10^−6^**	

RI: Retention Index.

**Table 3:** Inhibition zone diameters of bacterial (mm).

Essential Oils	Inhibition Zones in Diameters (mm) of Bacteria	
	*S. epidermidis*	*S. aureus*
Geranium oil	24.6	32.8
Tea Tree	20.5	39.1
*p*-value	0.070	

**Table 4:** Data are reported as ‘+’ indicates growth of *S. aureus* (not sensitive to tea tree oil and geranium essential oils), ‘-’ indicates inhibition of growth of bacteria (sensitive to tea tree and geranium essential oils).

Tea Tree Oil and Geranium Essential Oil on *S. aureus* and Control (μg/mL)	≥12.5	≥6.25	≥3.13	≥1.6	≥0.8	≥0.4	≥0.2	≥0.1
**Tea tree**	-	-	-	-	-	-	++	+++
**Geranium**	-	-	-	-	-	-	++	+++
**Control**	+++	+++	+++	+++	+++	+++	+++	+++

**Table 5:** Data are reported as ‘+’ indicates growth of *S. epidermis* (not sensitive to tea tree oil and geranium essential oils), ‘-’indicates inhibition growth of bacteria (sensitive to tea tree and geranium essential oils).

Tea Tree Oil and Geranium Essential Oil on *S. epidermis* and Control (μg/mL)	≥12.5	≥6.25	≥3.13	≥1.6	≥0.8	≥0.4	≥0.2	≥0.1
**Geranium**	-	-	-	-	-	-	-	++
**Tea tree**	-	-	-	-	-	-	+++	+++
**Control**	+++	+++	+++	+++	+++	+++	+++	+++

## Data Availability

The original contributions presented in this study are included in the article. Further inquiries can be directed to the corresponding author.

## References

[1] Oliveira W.F., Silva P.M.S., Silva R.C.S., Silva G.M.M., Machado G., Coelho L.C.B.B., Correia M.T.S. (2018). *Staphylococcus aureus* and *Staphylococcus epidermidis* infections on implants. J. Hosp. Infect..

[2] Rohde H., Burandt E.C., Siemssen N., Frommelt L., Burdelski C., Wurster S., Scherpe S., Davies A.P., Harris L.G., Horstkotte M.A. (2007). Polysaccharide intercellular adhesin or protein factors in biofilm accumulation of *Staphylococcus epidermidis* and *Staphylococcus aureus* isolated from prosthetic hip and knee joint infections. Biomaterials.

[3] Arciola C.R., Campoccia D., Montanaro L. (2018). Implant infections: Adhesion, biofilm formation, and immune evasion. Nat. Rev. Microbiol..

[4] Allizond V., Comini S., Cuffini A.M., Banche G. (2022). Current knowledge on biomaterials for orthopedic applications modified to reduce bacterial adhesive ability. Antibiotics.

[5] Dhar J., Thai A.L., Ghoshal A., Giomi L., Sengupta A. (2022). Self-regulation of phenotypic noise synchronizes emergent organization and active transport in confluent microbial environments. Nat. Phys..

[6] Gondil V.S., Subhadra B. (2023). Biofilms and their role in diseases. BMC Microbiol..

[7] Huang Z., Zhai Z., Zhou P., Li W., Hu W., Gong W. (2025). Antimicrobial Resistance and New Antimicrobial Agents: A Review of the Literature. Curr. Med. Chem..

[8] Khasapane N.G., Nkhebenyane J., Mnisi Z., Kwenda S., Thekisoe O. (2024). Comprehensive whole genome analysis of *Staphylococcus aureus* isolates from dairy cows with subclinical mastitis. Front. Microbiol..

[9] Salam M.A., Al-Amin M.Y., Salam M.T., Pawar J.S., Akhter N., Rabaan A.A., Alqumber M.A. (2023). Antimicrobial resistance: A growing serious threat to global public health. Healthcare.

[10] Chinemerem Nwobodo D., Ugwu M.C., Oliseloke Anie C., Al-Ouqaili M.T., Chinedu Ikem J., Victor Chigozie U., Saki M. (2022). Antibiotic resistance: The challenges and some emerging strategies for tackling a global menace. J. Clin. Lab. Anal..

[11] Setlhare G.G. (2017). An Investigation of Essential Oils as Antimicrobial Agents Against Antibiotic-resistant Bacteria Isolated at South African Hospices. Ph.D. Thesis.

[12] Liu J., Zhang X., Niu J., Han Z., Bi C., Mehmood K., Al Farraj D.A., Alzaidi I., Iqbal R., Qin J. (2023). Complete Genome of Multi-Drug Resistant Staphylococcus Aureus in Bovine Mastitic Milk in Anhui, China. Pak. Vet. J..

[13] Muhammad U.J., Muhammad I., Arslan A., Hamza R., Muhammad J.S., Ali A.J. (2024). Molecular Dynamics and Antimicrobial Resistance Pattern of β-lactam Resistant Coagulase Positive *Staphylococcus aureus* Isolated from Goat Mastitis. Pak. Vet. J..

[14] Sonola V.S., Misinzo G., Matee M.I. (2021). Occurrence of multidrug-resistant *Staphylococcus aureus* among humans, rodents, chickens, and household soils in Karatu, Northern Tanzania. Int. J. Environ. Res. Public Health.

[15] Iseppi R., Mariani M., Condò C., Sabia C., Messi P. (2021). Essential oils: A natural weapon against antibiotic-resistant bacteria responsible for nosocomial infections. Antibiotics.

[16] Ghavam M., Bacchetta G., Castangia I., Manca M.L. (2022). Evaluation of the composition and antimicrobial activities of essential oils from four species of *Lamiaceae Martinov* native to Iran. Sci. Rep..

[17] (2024). Nawzat Abozaid Issa, Evaluation the Antimicrobial Activity of essential oils against Veterinary Pathogens, Multidrug-resistant Bacteria and Dermatophytes. Pak. Vet. J..

[18] Mohamed A.E., Abdelrahman S.M., Mohamed A.H., Youssef H.A., Wafa S.M., Aljahdali A.O.S., Latifa A.H., Mohammed A.A., Mashail A.A., Amani O.A. (2024). Biochemical and Molecular Characterization of Five *Basil cultivars* Extract for Enhancing the Antioxidant, Antiviral, Anticancer, Antibacterial, and Antifungal Activities. Pak. Vet. J..

[19] Perna S., Alawadhi H., Riva A., Allegrini P., Petrangolini G., Gasparri C., Alalwan T.A., Rondanelli M. (2022). In vitro and in vivo anticancer activity of basil (*Ocimum* spp.): Current insights and future prospects. Cancers.

[20] Mohammed G., Abdulrahim R.H., Syeda S.S., Salma S., Tayyaba S., Waqas B., Azhar R. (2023). Antibacterial Activity of Aqueous and Methanolic Extract of *Mentha piperita* against Pervasive Bacteria Isolated from Urial the Ovis vignei. Pak. Vet. J..

[21] Swamy M.K., Akhtar M.S., Sinniah U.R. (2016). Antimicrobial Properties of Plant Essential Oils against Human Pathogens and Their Mode of Action: An Updated Review. Evid. Based Complement. Altern. Med..

[22] Gleń-Karolczyk K., Boligłowa E. (2015). Comparison of fungicidal properties of geranium and Tea tree oils. J. Res. Appl. Agric. Eng..

[23] Yadav E., Kumar S., Mahant S., Khatkar S., Rao R. (2017). Tea tree oil: A promising essential oil. J. Essent. Oil Res..

[24] Ren J., Wang Y.M., Zhang S.B., Lv Y.Y., Zhai H.C., Wei S., Ma P.A., Hu Y.S. (2024). Terpinen-4-ol from Tea tree oil prevents *Aspergillus flavus* growth in postharvest wheat grain. Int. J. Food Microbiol..

[25] Nguyen L., DeVico B., Mannan M., Chang M., Rada Santacruz C., Siragusa C., Everhart S., Fazen C.H. (2023). Tea tree Essential Oil Kills *Escherichia coli* and *Staphylococcus epidermidis* Persisters. Biomolecules.

[26] Celi D., Quiroz E., Beltrán-Noboa A., Machado A., Tejera E., Fernandez-Soto P. (2024). A chemical analysis of the *Pelargonium* species: *P. odoratissimum, P. graveolens*, and *P. zonale* identifies secondary metabolites with activity against gram-positive bacteria with multidrug-resistance. PLoS ONE.

[27] Bigos M., Wasiela M., Kalemba D., Sienkiewicz M. (2012). Antimicrobial activity of geranium oil against clinical strains of *Staphylococcus aureus*. Molecules.

[28] Oliva A., Costantini S., De Angelis M., Garzoli S., Božović M., Mascellino M.T., Vullo V., Ragno R. (2018). High potency of melaleuca alternifolia essential oil against multi-drug resistant gram-negative bacteria and methicillin-resistant *Staphylococcus aureus*. Molecules.

[29] Kirbaşlar F.G., Tavman A., Dülger B., Türker G. (2009). Antimicrobial activity of Turkish citrus peel oils. Pak. J. Bot..

[30] Kock J.L., Sebolai O.M., Pohl C.H., Van Wyk P.W., Lodolo E.J. (2009). Oxylipin studies Expose aspirin as antifungal. FEMS Yeast Res..

[31] Abidin Z.Z., Shamsudin N.S., Madehi N., Sobri S. (2013). Optimisation of a method to extract the active coagulant agent from *Jatropha curcas* seeds for use in turbidity removal. Ind. Crops Prod..

[32] Desam N.R., Al-Rajab A.J., Sharma M., Mylabathula M.M., Gowkanapalli R.R., Albratty M. (2019). Chemical constituents, in vitro antibacterial and antifungal activity of *Mentha× Piperita* L. (peppermint) essential oils. J. King Saud Univ.-Sci..

[33] Dzogbewu T.C. (2021). Laser powder bed fusion of Ti6Al4V-xCu: Process parameters. J. Met. Mater. Miner..

[34] Visan A.I., Negut I. (2024). Coatings based on essential oils for combating antibiotic resistance. Antibiotics.

[35] Ncango D.M., Swart C.W., Pohl C.H., Wyk P.V., Kock J.L. (2010). Mitochondrion activity and dispersal of *Aspergillus fumigatus* and *Rhizopus oryzae*. Afr. J. Microbiol. Res..

[36] Wingfield M.J., Van Wyk P.S. (1993). A new species of Ophiostoma from Protea infructescences in South Africa. Mycol. Res..

[37] Badr M.M., Taktak N.E., Badawy M.E. (2023). Comparison of the antimicrobial and antioxidant activities of tea tree (*Melaleuca alternifolia*) oil and its main component terpinen-4-ol with their nanoemulsions. Egypt. J. Chem..

[38] Kamel R., Afifi S.M., Abdou A.M., Esatbeyoglu T., AbouSamra M.M. (2022). Nanolipogel loaded with Tea tree oil for the management of burn: GC-MS analysis, in vitro and in vivo evaluation. Molecules.

[39] Fayoumi L., Khalil M., Ghareeb D., Chokr A., Bouaziz M., El-Dakdouki M.H. (2022). Phytochemical constituents and therapeutic effects of the essential oil of rose geranium (*Pelargonium hybrid*) cultivated in Lebanon. S. Afr. J. Bot..

[40] Yasin M., Younis A., Javed T., Akram A., Ahsan M., Shabbir R., Ali M.M., Tahir A., El-Ballat E.M., Sheteiwy M.S. (2021). River tea tree oil: Composition, antimicrobial and antioxidant activities, and potential applications in agriculture. Plants.

[41] Abdelhamed F.M., Abdeltawab N.F., ElRakaiby M.T., Shamma R.N., Moneib N.A. (2022). Antibacterial and anti-inflammatory activities of *Thymus vulgaris* essential oil nanoemulsion on acne vulgaris. Microorganisms.

[42] Mahboubi M. (2016). Rosa damascena as holy ancient herb with novel applications. J. Tradit. Complement. Med..

[43] Hsouna A.B., Hamdi N. (2012). Phytochemical composition and antimicrobial activities of the essential oils and organic extracts from Pelargonium graveolens growing in Tunisia. Lipids Health Dis..

[44] Elghali F., Ibrahim I., Guesmi M., Frikha F., Mnif S. (2024). Unveiling the impact of selected EO’s on MRSA strain ATCC 33591: Antibacterial efficiency, biofilm disruption, and staphyloxanthin inhibition. Braz. J. Microbiol..

[45] Khatoon Z., McTiernan C.D., Suuronen E.J., Mah T.F., Alarcon E.I. (2018). Bacterial biofilm formation on implantable devices and approaches to its treatment and prevention. Heliyon.

[46] Zainal Abidin M., Shamsuddin R., Othman Z., Abdul Rahman R. (2013). Effect of postharvest storage of whole fruit on physico-chemical and microbial changes of fresh-cut cantaloupe (*Cucumis melo* L. reticulatus cv. Glamour). Int. Food Res. J..

[47] Malizos K.N., Kirketerp-Møller K. (2016). Incidence and socioeconomic impact of Bone and Joint Infections (BJIs): The European perspective. Periprosthetic Joint Infections: Changing Paradigms.

[48] Magunga B.T. (2016). An Investigation of Alternative Antifungals against *Phyllosticta citricarpa* Kiely and *Guignardia mangiferae*. Ph.D. Dissertation.

[49] Ntondini S.S., Lenetha G., Dzogbewu T.C. (2021). Antimicrobial Activity of *Salvia Officinalis* against Streptococcus Mutans Causing Dental Implant Failure: An in vitro Study. Int. Oral Health.

[50] Al Sevik R., Akarca G., Kilinc M., Ascioglu Ç. (2021). Chemical composition of Tea tree (*Melaleuca alternifolia*)(Maiden & Betche) cheel essential oil and its antifungal effect on foodborne molds isolated from meat products. J. Essent. Oil Bear. Plants.

